# Die Berliner Stadtreinigung (BSR) auf dem Weg ins „Neue Normal“ – Eine Fallstudie

**DOI:** 10.1007/s11613-022-00795-y

**Published:** 2022-11-17

**Authors:** Stefanie Hansen-Heidelk

**Affiliations:** 1 Institut für Psychologie, Universität Kassel, Holländische Straße 36–38, 34127 Kassel, Deutschland; 2 BSR, Ringbahnstraße 96, 12103 Berlin, Deutschland

**Keywords:** Homeoffice, Hybride Arbeitsorganisation, Neues Normal, Home office, Hybrid work organization, New normal

## Abstract

Die COVID-19-Pandemie war der größte Transformationsbeschleuniger für die Arbeitsorganisation. Die BSR hat in einer repräsentativen Studie 2374 Beschäftigten zu Erfahrungen und Erwartungen mit mobilen Arbeiten (MoA) befragt. Es konnten keine unterschiedlichen Erwartungen bei Beschäftigten verschiedener Verwaltungsbereiche oder unterschiedlicher Altersgruppen nachgewiesen werden. Auch die familiäre Situation führt zu keinen anderen Erwartungen an das MoA. Führungskräfte wünschen sich weniger MoA. Die meisten wollen 2 bis 4 Tage im MoA arbeiten. Es bedarf daher keiner differenzierten Regelungen für unterschiedliche Beschäftigtengruppen für das langfristige Gelingen des mobilen Arbeitens.

## Abstract (en)

The COVID 19 pandemic was the biggest transformation accelerator for work organization. In a representative study, BSR asked 2374 employees about their experiences and expectations with mobile working (MoA). No different expectations among employees in different administrative areas or different age groups could be demonstrated. Family situation also does not lead to different expectations of MoA. Managers want less MoA. Most want to work 2 to 4 days in MoA. Therefore, there is no need for differentiated arrangements for different employee groups for the long-term success of mobile working.

## Einleitung

Die Berliner Stadtreinigung (BSR) ist eine Anstalt des öffentlichen Rechts des Landes Berlin und mit rund 6000 Beschäftigten das größte kommunale Stadtreinigungs- und Entsorgungsunternehmen Deutschlands. Etwas mehr als ein Drittel der Beschäftigten arbeitet überwiegend oder zumindest teilweise an Büroarbeitsplätzen. Vor der COVID-19-Pandemie stellte das mobile ortsunabhängige Arbeiten (MoA)^1^ die absolute Ausnahme dar. Die Pandemie hat die Arbeitssituation der Beschäftigten mit Büroarbeitsplätzen in der BSR schlagartig verändert. Ab März 2020 haben bis zu 1000 Beschäftigte pro Tag zumindest teilweise im MoA gearbeitet. Dies entspricht der Situation von Millionen von Beschäftigten in Deutschland. Vor der Pandemie lag die Quote des mobilen Arbeitens zwischen 12 und 22 % (Bonin et al. 2020) und ist während der Pandemie auf bis zu 51 % angestiegen (Bonin et al. 2021; Frodermann et al. 2020).

Eine solch rasante Umstellung der Arbeitsorganisation für eine so große Beschäftigtengruppe hat es in dieser Form bislang selten gegeben. Die Erfahrungen der Beschäftigten, Führungskräfte und Unternehmen mit MoA konnten durch diese Ausnahmesituation umfangreich untersucht wurden (Boockmann et al. 2021; Flüter-Hoffmann und Stettes 2022). Damit wurde Deutschland quasi zu einem großen Experimentierraum für ein „Neues Normal“ in der Arbeitsorganisation (Hofmann et al. 2020). Viele Beschäftigte haben gute Erfahrungen mit dem mobilen Arbeiten gemacht, und rund zwei Drittel wollen auch nach der Pandemie an einigen Tagen weiterhin mobil arbeiten (Bonin et al. 2020). Das stellt die Unternehmen vor große Herausforderungen. Einerseits bieten mobile Arbeitsformen Vorteile für viele Beschäftigte wie bessere Vereinbarkeit von Beruf und Familie, reduzierte Arbeitswege, mehr Selbstbestimmung und höhere Arbeitszufriedenheit, Effizienz und Produktivität (Kunze et al. 2020). Andererseits birgt die Flexibilisierung auch Risiken wie z. B. fragmentiertes Arbeiten über den Tag und die Woche verteilt, Vereinsamung und psychische Überlastung (Berzel und Schroeder 2021). Jeder fünfte Betrieb will das mobile Arbeiten ausbauen (Bellmann et al. 2021), und immerhin 60 % der Betriebe sehen keinen Unterschied in der Produktivität der Beschäftigten (IAB 2021).

Diese Vor- und Nachteile des mobilen Arbeitens gilt es unter gleichzeitiger Berücksichtigung der Interessen der Beschäftigten und der Betriebe auszugestalten. Um mobiles Arbeiten langfristig erfolgreich zu implementieren und in die analoge Arbeitswelt zu integrieren, muss die Arbeitsorganisation grundlegend neugestaltet werden. Einige Studien, die während der COVID-19-Pandamie entstanden sind, zeigen weitere Faktoren auf, die beim langfristigen Gelingen von mobilen Arbeitsformen berücksichtigt werden sollten. Die Unternehmenskulturen müssen sich weg von Präsenz- zu Homeoffice-Kulturen entwickeln, die durch starkes Vertrauen in die Beschäftigten, eine wertschätzende Kommunikation sowie Regeln über die Erreichbarkeit gekennzeichnet sind (Neumann et al. 2020). Der zentralen Herausforderung des Arbeitens und Führens auf Distanz (Avermeyer et al. 2022) muss durch vertrauensbildende Maßnahmen begegnet werden (Jäckel 2020). Zudem müssen die Beschäftigten und Führungskräfte befähigt werden, die neuen Anforderungen zu bewältigen (Mander et al. 2021).

Vor diesem Hintergrund wurden die Beschäftigten der BSR befragt, welche Erfahrungen sie während der COVID 19-Pandemie mit dem mobilen Arbeiten gemacht haben und welche Erwartungen sie an das „Neue Normal“, der Arbeitsorganisation der Zukunft haben. Anhand der Ergebnisse der Befragung und der Berücksichtigung der weiteren Studienergebnisse werden Rahmenbedingungen zur bestmöglichen Gestaltung der Arbeitsorganisation der Zukunft für die BSR abgeleitet.

## Fragestellungen und Hypothesen

### Fragestellungen

1. Welche Erfahrungen und welche Erwartungen haben die Beschäftigten der BSR differenziert nach Altersgruppen, Tätigkeitsbereichen, familiärer Situation sowie Beschäftigten mit und ohne Führungsverantwortung an das MoA?
2. Wie kann eine neue Arbeitsorganisation unter Integration des MoA für die BSR aussehen, die die Erwartungen der Beschäftigten ausreichend berücksichtigt?

### Hypothesen

#### H1

Es gibt keine Unterschiede in den Erwartungen der Beschäftigten an das MoA zwischen den einzelnen Geschäftsbereichen der zentralen Verwaltung.

#### H2

Die Beschäftigten mit zu betreuenden Kindern oder anderen Familienangehörigen wünschen sich eine intensivere Nutzung des MoA als die Beschäftigten ohne Betreuungsverantwortung.

#### H3

Die jüngeren Beschäftigten wünschen sich eine intensivere Nutzung des MoA als die Älteren.

#### H4

Führungskräfte wünschen eine geringere Nutzung des MoA als Beschäftigte ohne Führungsverantwortung.

## Methodik

Die Erfahrungen und Erwartungen der Beschäftigten an das MoA wurden durch eine quantitative anonyme Befragung in der BSR erhoben. Der Fragebogen, der in 3 Abschnitte gegliedert ist, wurde in einer Arbeitsgruppe bestehend aus Vertretenden verschiedener Beschäftigtengruppen und Interessenvertretungen sowie der Begleitung durch die Syspons GmbH entwickelt. In Abschn. 1 wurden personenbezogene Fragen wie z. B. Alter, Tätigkeitsdauer in der BSR, Geschäftseinheit, Betreuung von Familienangehörigen und Führungsverantwortung gestellt. In Abschn. 2 wurden die Erfahrungen mit MoA wie z. B. Rahmenbedingungen für MoA, Einschätzung von MoA im Vergleich zu analogem Arbeiten und subjektive Beurteilung des MoA. Die Einschätzung erfolgte anhand einer Likert-Skala von 1 bis 6 bzw. bei den Fragen zu den Auswirkungen des MoA anhand einer Likert-Skala von − 2 bis + 2. Sieben Fragen zu den Erwartungen an das MoA in der Zukunft wurden in Abschn. 3 gestellt. Unter anderem wurde z. B. nach dem Stellenwert von MoA in der Zukunft, den Aktivitäten, die weiterhin im Büro ausgeführt werden sollen, der Ausgestaltung der Arbeit im Team und dem Umfang des MoA im Arbeitsalltag gefragt. Die Befragung wurde durch die Syspons GmbH digital mit dem Befragungstool LimeSurvey von Februar bis März 2022 durchgeführt. Es wurden alle Beschäftigten der BSR, die über eine betriebsinterne E‑Mail-Adresse verfügen, einbezogen. Die Daten wurden anonymisiert gespeichert.

Die deskriptive Statistik der Ergebnisse erfolgte durch Darstellung der Mittelwerte mit Standardabweichungen (MW ± SD) sowie im Falle der skalierten Fragen ergänzend durch die Darstellung der Verteilung der Antworten auf die einzelnen Bewertungen.

Die Daten wurden zunächst mittels Shapiro-Wilk-Test auf Normalverteilung geprüft. Zur Überprüfung der Hypothesen 1 und 3 wurden die Daten zunächst mit Hilfe des Levene-Tests auf Varianzhomogenität geprüft. Bei Varianzhomogenität wurden Unterschiede zwischen den Gruppen mittels Varianzanalyse auf Signifikanz geprüft. Bei fehlender Varianzhomogenität kam der Welch-Test zur Anwendung. Als post-hoc Tests zur Überprüfung von Unterschieden zwischen den Gruppen wurde der Tukey-Test bei Varianzhomogenität bzw. der Games-Howell-Test bei fehlender Varianzhomogenität eingesetzt. Zur Überprüfung der Hypothesen 2 und 4 wurden die Daten zunächst mittels Shapiro-Wilk-Test auf Normalverteilung geprüft. Unterschiede zwischen den Gruppen wurden mittels t‑Test für unabhängige Stichproben auf Signifikanz geprüft. Bei fehlender Normalverteilung kam der Mann-Whitney U‑Test zur Anwendung. Signifikanz wurde bei einem *p* < 0,05 angenommen. Da die Aussagekraft des parametrischen und des nicht-parametrischen Tests bei Likert-Skalen vergleichbar ist (de Winter und Dodou 2010) und die Aussagekraft der Mediane mit Interquartilsabstand bei skalierten Variablen begrenzt ist, werden alle Ergebnisse als Mittelwerte mit Standardabweichung dargestellt.

## Ergebnisse

Es wurden 2374 Mitarbeitenden angeschrieben, 881 Beschäftigten sind davon den zentralen Verwaltungsbereichen zuzuordnen, 1493 den operativen Bereichen. Insgesamt haben 981 Beschäftigte, entsprechend 43,3 %, den Fragebogen beantwortet. Die Rücklaufquote in den einzelnen Geschäftseinheiten der zentralen Verwaltung mit Ausnahme der Auszubildenden und Dual-Studierenden lag sogar zwischen 68–98 %, bei den 1493 Beschäftigten der operativen Bereiche bei 28 % (*n* = 418). 54 % der Befragten waren Frauen, 46 % Männer. 8 % aller Befragten war für die Betreuung von Kindern bzw. Angehörigen allein zuständig, 32 % gemeinsam mit dem/der Partner:in. Entsprechend 60 % hatten keine Betreuungsverantwortung. 257 (25,2 %) der Befragten hatten Führungsverantwortung, wobei 11,8 % der 4. Ebene, 9,5 % der 3. Ebene und 3,9 % der 2. Führungsebene angehörten.

### Gesamtergebnisse

**Figure 1:**
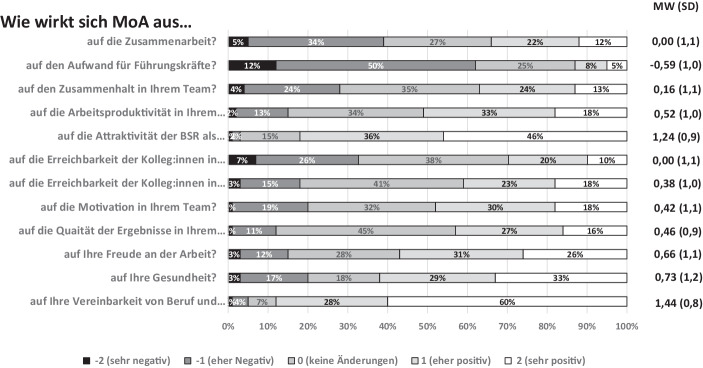

Insgesamt gaben 79 % der Befragten an, im MoA gearbeitet zu haben. 60 % haben fast ausschließlich, 19 % etwa hälftig im MoA und im Büro gearbeitet. 21 % waren überwiegend im Büro. Die subjektive Einschätzung der Befragten wie gut das Arbeiten im MoA geklappt hat, wurde im Mittel mit 5,1 (1,0) als sehr gut bewertet. Die Einschätzung der Auswirkungen des MoA auf die Zusammenarbeit und wichtige Rahmenbedingungen des Arbeitens erfolgte auf einer Skala von − 2 (sehr negativ) bis + 2 (sehr positiv). Eine übersichtliche Ergebnisbewertung stellt die Betrachtung der Mittelwerte dar: Ein Mittelwert von 0 zeigt keine Auswirkungen, ein Mittelwert > 0 eine positive Auswirkung des MoA, ein Mittelwert < 0 entsprechend eine negative Auswirkung an. Sehr positiv wurde die Vereinbarkeit von Beruf und Familie im MoA sowie die Attraktivität des Arbeitgebers bewertet. Die Auswirkung auf die Gesundheit, die Arbeitsproduktivität und die Freude am Arbeiten wurden als eher positiv im Vergleich zur Arbeit in Präsenz bewertet. Negativ hingegen wurde von den Führungskräften der Aufwand für Führungsaufgaben beurteilt (Abb. 1).

**Figure 2:**
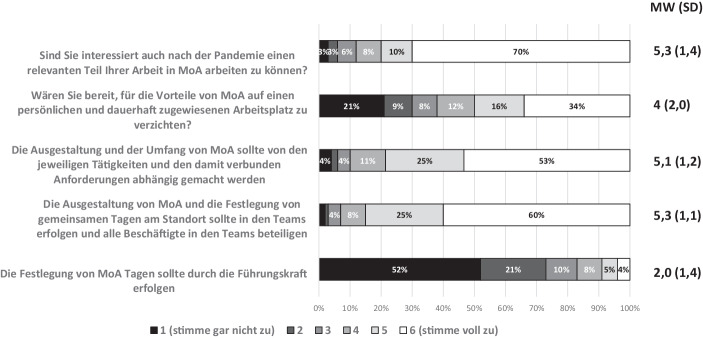

In mehreren Fragen wurde die Einschätzung zur Relevanz des mobilen Arbeitens für die zukünftige Arbeitsorganisation in der BSR evaluiert. Der Mittelwert bezüglich des Interesses, auch zukünftig einen relevanten Anteil in MoA arbeiten zu können, lag mit 5,3 (1,4) sehr hoch. Ein Großteil der Befragten wäre sogar dazu bereit, auf einen persönlich und dauerhaft zugewiesenen Arbeitsplatz in der BSR zu verzichten (MW 4 (2)). Bei der Frage zur Ausgestaltung des MoA haben die meisten Beschäftigten eine klare Vorstellung, wie diese aussehen sollte. Mit einem Mittelwert von 5,1 (1,2) sind die Beschäftigten der Auffassung, dass der Umfang des MoA von den Tätigkeiten und den damit verbundenen Aufgaben abhängig gemacht werden sollte. Mit ebenfalls sehr hoher Zustimmung von im Mittel 5,3 (1,1) waren die Beschäftigten der Auffassung, dass die Festlegung von gemeinsamen Tagen im Büro von allen Beschäftigten gemeinsam im Team erfolgen sollte. Eine Festlegung der MoA-Tage durch Führungskräfte wurde mit einer mittleren Bewertung von 2 (stimme nicht zu) deutlich abgelehnt (Abb. 2).

Die Anzahl der gewünschten MoA-Tage in Zukunft gaben die Beschäftigten recht einheitlich mit 2,8 (1,3) an. 73 % wünschen sich zwischen 2 und 4 Tage MoA pro Woche. Damit korrespondiert die gewünschte Anzahl von Mindesttagen im Büro an BSR-Standorten mit 1,6 (1,1) Tagen pro Woche.

### Analyse unterschiedlicher Gruppen Beschäftigter

Für die Analyse der unterschiedlichen Subgruppen, auf die sich die Hypothesen beziehen, wurden die Fragen aus dem Abschn. 3 des Fragebogens „Erwartungen der Beschäftigten an das MoA in der Zukunft“ ausgewertet.

#### Geschäftsbereiche

Um zu prüfen, ob die Beschäftigten verschiedener Geschäftsbereiche unterschiedliche Erwartungen an das MoA haben, wurden die Ergebnisse der Befragung zwischen den zentralen und operativen Geschäftsbereichen verglichen. Die große Gruppe der operativen Bereiche umfasst die Müllabfuhr, Straßenreinigung, Anlagen, Fuhrparkmanagement, Betriebsgastronomie und das Immobilienmanagement. Die Analysen ergaben mit Ausnahme der beiden Fragen zur Ausgestaltung des MoA signifikante Unterschiede zwischen den Geschäftsbereichen. Allerdings waren diese Unterschiede nur zwischen den zentralen Geschäftsbereichen und den operativen Bereichen nachweisbar. Bei nahezu allen Erwartungen wünschten sich die Beschäftigten der operativen Bereiche im Vergleich zu den zentralen Geschäftsbereichen signifikant weniger MoA, was sich insbesondere an den mit 2,5 (1,3) geringeren MoA-Tagen pro Woche und mit 2,1 (1,2) nahezu doppelt so vielen Präsenztagen an der BSR zeigt.

Zwischen den zentralen Geschäftsbereichen hingegen konnten bei sehr hohen mittleren Zustimmungswerten keine Unterschiede nachgewiesen werden. Das Interesse an MoA war sehr hoch ebenso wie die Bereitschaft, auf einen festen Arbeitsplatz zu verzichten. Einheitlich wurden 2,9 bis 3,4 Tage pro Woche MoA gewünscht und im Mittel lediglich 1,0–1,3 Tage Präsenz an einem BSR-Standort. Für die meisten gefragten Erwartungen konnten keine signifikanten Unterschiede zwischen den einzelnen zentralen Geschäftsbereichen nachgewiesen werden. Damit konnte die Hypothese 1 bestätigt werden.

#### Beschäftigte mit und ohne Betreuung von Kindern bzw. anderen Angehörigen

Zur Prüfung der Hypothese 2, dass es Unterschiede in den Erwartungen an das MoA in der zukünftigen Arbeitsorganisation zwischen Beschäftigten mit (*n* = 367) und ohne (*n* = 558) zu betreuende Kinder oder Familienangehörige gibt, wurden die Erwartungen zwischen diesen beiden Gruppen verglichen. Es konnte lediglich für die Bereitschaft, auf einen individuellen Arbeitsplatz an einem BSR-Standort zu verzichten, ein signifikanter Unterschied zwischen den beiden Gruppen nachgewiesen werden. Die Beschäftigten mit Betreuungsaufgaben sind mit einem Mittelwert von 4,4 etwas häufiger bereit, auf einen festen Arbeitsplatz zu verzichten, im Vergleich zu 3,7 bei Beschäftigten ohne Betreuungsaufgaben. Bei den anderen erfragten Erwartungen an das MoA konnten überraschenderweise keine Unterschiede zwischen den beiden Gruppen nachgewiesen werden. Damit musste die Hypothese 2 abgewiesen werden.

#### Unterschiedliche Altersgruppen

Grundsätzlich waren die Erwartungen an das MoA zwischen den einzelnen Altersgruppen einheitlich. Allerdings konnten für die Altersgruppe der 25–34-Jährigen signifikante Unterschiede insbesondere gegenüber den Altersgruppen ab 45 Jahre nachgewiesen werden. So war das Interesse an MoA sowie die Bereitschaft, auf einen festen Arbeitsplatz zu verzichten, in dieser Altersgruppe höher. Die Anzahl der gewünschten MoA-Tage pro Woche lag mit 3,4 ebenfalls höher als in den anderen Altersgruppen mit 2,6–2,9 Tagen. Die Altersgruppen < 35 Jahre erwarteten etwas weniger deutlich die Festlegung der Ausgestaltung und des Umfangs von MoA durch die Teams. Allerdings waren diese Unterschiede gegenüber den älteren Beschäftigten gering. Die Festlegung der gemeinsamen Tage wurde in allen Altersgruppen mit Werten zwischen 5,1 und 5,4 durch die Teams gewünscht. Somit konnte die Hypothese 3 bestätigt werden.

#### Beschäftigte mit Führungsverantwortung

In die Gruppe Führungskräfte wurden alle Beschäftigten mit Führungsverantwortung aller Ebenen aufgenommen (*n* = 245). 741 Befragte hatten keine Führungsverantwortung. In nahezu allen Fragen zum zukünftigen MoA unterscheiden sich die Führungskräfte signifikant von den Beschäftigte ohne Führungsverantwortung. Das Interesse an MoA ist etwas geringer, und auch auf einen festen Arbeitsplatz wollten Führungskräfte seltener verzichten. Der Umfang des MoA soll nach Auffassung der Führungskräfte mit 2,2 Tagen pro Woche weniger umfangreich sein, als es die Beschäftigten ohne Führungsverantwortung im Mittel 3,0 Tagen wünschen. Entsprechend wünschen die Führungskräfte mehr Präsenztage an einem BSR-Standort. Bei der Festlegung der MoA-Tage wollen Führungskräfte diese etwas häufiger selbst festlegen, allerdings ist der Wunsch mit einem Wert von 2,5 auf der Skala von 1 bis 6 insgesamt wenig ausgeprägt. Insgesamt konnte damit die Hypothese 4 bestätigt werden.

## Diskussion

Die hohe Beteiligung an der Befragung von 43 % insgesamt und über 90 % in den zentralen Geschäftsbereichen zeigt das große Interesse der Beschäftigten, auch zukünftig mobil arbeiten zu können.

### Erfahrungen mit MoA

Mit insgesamt 79 % ist die Quote der Beschäftigten in der BSR, die während der Covid19-Pandemie teilweise oder ausschließlich im MoA gearbeitet haben, deutlich höher als in anderen Studien. Dort werden Quoten von 42 % bis maximal 51 % angegeben (Bonin und Rinne 2021; Frodermann et al. 2020). Für diese sehr hohe Homeoffice-Quote in der BSR sind verschiedene Ursachen zu diskutieren. Ein wesentlicher Unterschied dürfte in der Zusammensetzung der Gruppe der Befragten liegen. In der vorliegenden Studie wurden nur die Beschäftigten mit dienstlicher E‑Mail-Adresse einbezogen, die mindestens teilweise ihre Tätigkeiten mobil erledigt können. Ein weiterer Grund für die intensive Nutzung des MoA könnte eine sehr hohe Quote an ausreichender technischer Ausstattung der MoA-Arbeitsplätze sein, da lediglich 3,5 % angegeben haben, über keine ausreichenden technischen Möglichkeiten für ihre Tätigkeiten im MoA zu verfügen. In anderen Studien liegt diese Rate deutlich höher (Frodermann et al. 2021). Von den Beschäftigten, die nicht im MoA gearbeitet haben, gaben 79 % als Haupthinderungsgrund die Nichteignung der Tätigkeit für Homeoffice an. Auch diese Quote lag in einer anderen Studie mit 84 % der Befragten etwas höher (ebd). Identisch war die Häufigkeit des Hinderungsgrundes, Beruf und Privatleben schlechter trennen zu können, mit 18,5 % in der BSR bzw. 18 % bei Frodermann et al. (2021). Im Gegensatz dazu haben nur 10 % der befragten BSR-Beschäftigten mangels Unterstützung durch die Führungskraft nicht in MoA gearbeitet, während bei Frodermann et al. (2021) die Quote mit 17 % deutlich höher lag.

79 % der befragten Beschäftigten beurteilen die bisherige Arbeit im MoA als gut bis sehr gut. Auch diese Quote liegt deutlich über den Ergebnissen anderer Befragungen, die z. B. mit 61 % angegeben werden (ebd.). Das Arbeiten im MoA wurde von den Beschäftigten für die meisten gefragten Aspekte eher positiv im Vergleich zur Präsenztätigkeit vor der Pandemie gesehen; so wurde z. B. die Arbeit im MoA als effizienter eingeschätzt. Der Mittelwert lag bei 0,52, was nahezu identisch mit Ergebnissen anderer Befragungen wie der IAB-Studie ist, die einem Wert von 0,43 ermittelt hat (ebd.). Beide Studien haben eine Skala von − 2 bis + 2 verwendet.

Ein wichtiges Ergebnis war die sehr positive Bewertung der Arbeitgeberattraktivität durch das MoA. Damit bestätigen die Beschäftigten der BSR, dass das MoA-Angebot mittlerweile ein wichtiger Faktor bei der Arbeitsplatzwahl ist und mit einem erfolgreich umgesetzten flexiblen Arbeitsorganisationskonzept Wettbewerbsvorteile zu erzielen sind. Das liegt sogar über den Ergebnissen der Studie von Backhaus et al. (2020), wonach aus Sicht der befragten Betriebe das Angebot von Homeoffice die Arbeitgeberattraktivität nur um 54 % steigert. Auch wenn 62 % meinen, dass das Arbeiten im MoA sich positiv bis sehr positiv auf ihre Gesundheit auswirkt, sollte das Thema Gesundheit bei der zukünftigen Gestaltung von MoA sorgfältig beobachtet werden, zumal nach Hofmann et al. (2020) 40 % der befragten Unternehmen angaben, dass Beschäftigte über Haltungsprobleme wie Rückenschmerzen klagen. Auch lässt diese Befragung keine Aussagen auf die psychische Gesundheit zu, da dieser Aspekt nicht Gegenstand der Befragung war. In der Studie von Kunze et al. (2020) wurde gezeigt, dass emotionale Erschöpfung und das Gefühl sozialer Isolation im Homeoffice für 16 % bzw. 20 % wichtige Themen sind. Mit 88 % war der Wert der Befragten, die Vereinbarkeit von Beruf und Familie durch MoA als positiv bis sehr positiv eingeschätzt haben, fast identisch mit dem Ergebnis der Studie von Ahlers et al. (2021).

### Erwartung an die zukünftige Arbeit mit MoA insgesamt

Mit 80 % möchte ein Großteil der befragten Beschäftigten auch weiterhin einen relevanten Teil der Arbeitszeit in MoA arbeiten. Das entspricht auch den Ergebnissen anderer Studien (IG Metall 2020; Bonin et al. 2021). Überraschend war die hohe Bereitschaft von ca. 50 %, auf einen festen persönlichen Arbeitsplatz zu verzichten. 78 % meinen, dass die Möglichkeit zur Nutzung von MoA von den Tätigkeiten abhängig sein sollte, und nach Auffassung von 85 % sollte die Ausgestaltung in den jeweiligen Teams erfolgen. Eine Festlegung durch die Führungskräfte wird überwiegend abgelehnt. Diese eindeutigen Ergebnisse zeigen den Wunsch nach Selbstgestaltung und Mitwirkung bei der Arbeitsorganisation. Die Frage nach der Anzahl der gewünschten Tage im MoA wurde im Mittel mit 3 Tagen beantwortet. Dabei war die Spreizung erstaunlich gering, 79 % aller Beschäftigten wünschen sich zwischen 2 und 4 Tagen MoA pro Woche und entsprechend 1–2 Tage Präsenz im Büro. Dieses klare Ergebnis ist homogener als die Ergebnisse anderer Befragungen. So konnten Kunze et al. (2020) zeigen, dass 54 % der Befragten 2–4 Tage und 25 % sogar 5 Tage pro Woche im Homeoffice arbeiten wollen.

### Erwartungen einzelner Beschäftigtengruppen

Es gab keine signifikanten Unterschiede in den Erwartungen der Beschäftigten an das MoA zwischen den zentralen Geschäftsbereichen. Bisher wurden Unterschiede in der Nutzung des MoA nur in verschiedenen Branchen untersucht, die Nutzung in verschiedenen Dienstleistungsbereichen eines Unternehmens lediglich in der Befragung der IG Metall, die ebenfalls keine relevanten Unterschiede in der Nutzung des MoA aufzeigen konnte (IG Metall 2020). Dieses Ergebnis erleichtert die Konzeption einer neuen Arbeitsorganisation, da es somit einheitliche Rahmenregelungen für alle Beschäftigten über alle Geschäftsbereiche hinweg geben kann.

Überraschend war das Ergebnis, dass in der BSR keine relevanten Unterschiede in den Erwartungen an das MoA zwischen Beschäftigten mit und ohne Betreuung von Angehörigen – in der Regel von Kindern – nachgewiesen werden konnten. Zwar wurde mit einem mittleren Wert von 5,4 etwas stärker eine zukünftige Tätigkeit im MoA von Beschäftigten ohne Betreuungsaufgabe mit 5,2 gewünscht. Dieser Unterschied ist aber sehr gering und nicht relevant. Die Bereitschaft, auf einen individuell zugewiesenen Arbeitsplatz zu verzichten, war bei Beschäftigten mit zu betreuenden Angehörigen höher im Vergleich zu Beschäftigten ohne Betreuungsverantwortung. Da aber die Bereitschaft insgesamt sehr hoch war, ist dieser signifikante Unterschied wenig relevant. Bei allen anderen Vorstellungen über die gerechte Vereinbarung zur Nutzung des MoA gab es keinen Unterschied zwischen Beschäftigten mit und ohne Betreuungsaufgaben. Beschäftigte der BSR mit zu betreuenden Angehörigen erwarten offensichtlich keine erweiterte Flexibilität oder Sonderregelungen.

Zwischen den Altersgruppen gab es nur geringe Unterschiede hinsichtlich der Erwartung an MoA in der Zukunft. Die Altersgruppe der 25–34-Jährigen hatte eine etwas höhere Bereitschaft, auf einen Arbeitsplatz zu verzichten, und wünschte sich gegenüber der ältesten Beschäftigtengruppe etwas mehr MoA-Tage und etwas weniger Tage im Büro. Dieses Ergebnis bestätigt die Befragung der IG Metall, in der ebenfalls gezeigt wurde, dass die Altersgruppen 25–34 und 35–44 mit jeweils etwa 90 % sich häufiger regelmäßiges Arbeiten von zu Hause wünschen als die übrigen Altersgruppen mit 70–85 % (IG Metall 2020). Bei den weiteren Fragen zu den Erwartungen an das MoA in Zukunft gab es keine Unterschiede zwischen den Altersgruppen.

Im Gegensatz dazu konnte die Hypothese eines Unterschieds der Erwartungen zwischen Beschäftigten mit und ohne Führungsverantwortung in der Befragung bestätigt werden. Die Führungskräfte haben zwar ebenfalls ein hohes Interesse an MoA, dieses war aber genauso wie die Bereitschaft, auf einen festen Arbeitsplatz zu verzichten, etwas geringer als bei den übrigen Beschäftigten. Auch die Anzahl der gewünschten MoA-Tage war um knapp 1 Tag niedriger und entsprechend die Anzahl der gewünschten Bürotage mit 2,2 vs. 1,4 Tage/Woche um knapp 1 Tag pro Woche höher. Auch bei der Ausgestaltung des MoA waren die Führungskräfte häufiger der Meinung, dass dieses durch die Führungskräfte festgelegt werden sollte. Hinzu kam die bereits oben erwähnte Einschätzung von 62 % der befragten Führungskräfte, dass sich der Führungsaufwand erhöhen würde. Dies entspricht auch den Ergebnissen anderer Befragungen (Hofmann et al. 2020). So stimmten zwar mehr als 50 % der Befragten der Aussage zu, dass durch den vermehrten Einsatz von Homeoffice Vorbehalte bei Führungskräften reduziert, aber nicht gänzlich abgebaut werden konnten. Als Gründe hierfür werden in der Studie die Präsenzorientierung und die ungenügende Betreuung durch die Führungskräfte genannt.

### Limitationen der Befragungsergebnisse

Es wurden nur die Beschäftigten befragt, die über eine betriebsinterne E‑Mail-Adresse verfügen. Da dies insbesondere in den operativen Bereichen bei deutlich unter 50 % der Befragten der Fall ist, ist die Repräsentativität der Ergebnisse hier limitiert. Weiterhin ist die Vergleichbarkeit der Ergebnisse mit denen anderer Befragungen durch methodische Unterschiede eingeschränkt. Insbesondere die Möglichkeiten der Antworten anhand einer Likert-Skala von 1 bis 6 unterscheiden sich von z. B. ja – nein Antwortmöglichkeiten in anderen Studien. Auffallend war auch eine fehlende Normalverteilung vieler Ergebnisse, die durch eine Likert-Skala erhoben wurden. Häufig war eine ausgeprägte Verschiebung nach rechts oder links nachweisbar. Dieses lässt vermuten, dass die Befragten eher entsprechend einer ja – nein Frage geantwortet haben. Es könnte durch die Formulierung der Fragen mit bedingt sein. Letztlich wurden keine Fragen zur Unterstützung der Beschäftigten bei der dauerhaften Implementierung eines „neuen Normal“ gestellt.

Die nachfolgend dargestellten Handlungsempfehlungen für die praktische Umsetzung von MoA beruhen auf den Ergebnissen dieser Arbeit. Die zur Umsetzung zusätzlich erforderlichen unterstützenden Maßnahmen fußen dagegen nur auf einzelnen Erkenntnissen aus den Studien, die sich mit Fragestellung hierzu beschäftigt haben.

## Handlungsempfehlungen für die praktische Umsetzung von MoA

Aus den Befragungsergebnissen und den Erkenntnissen von Studien lassen sich Empfehlungen ableiten, die für das Gelingen einer neuen Arbeitsorganisation in der BSR, dem „Neuen Normal“, entscheidend sind.

### Rahmenbedingungen für flexible Anzahl an MoA-Tagen schaffen

Es sollten Rahmenbedingungen in der BSR geschaffen werden, die es ermöglichen, dass ein relevanter Anteil der Arbeitszeit (2 bis 4 Tage) im MoA gearbeitet werden kann, wenn die Tätigkeiten im MoA ausgeübt werden können und die Produktivität dadurch nicht eingeschränkt wird. Die Arbeitsorganisation der jeweiligen Arbeitsbereiche ist dabei zu berücksichtigen. In der BSR werden bereits verschiedene Modelle mit unterschiedlich vielen Präsenz- und MoA-Tagen ausprobiert. Aus den Erfahrungen sollen Rahmenbedingungen für die BSR insgesamt in einer Dienstvereinbarung festgelegt werden.

### Präsenztage für das Arbeiten im Betrieb festlegen

Für die gemeinsame Arbeit im Büro sollten 1–2 Präsenztage pro Woche für Besprechungen und regelmäßige Termine, den sozialen Kontakt, den Austausch und die Projekt- und Teamarbeit festgelegt werden. Die soziale Vernetzung, die durch die Homeoffice-Situation erschwert ist, muss durch digitale Formate und Präsenzveranstaltungen aktiv von der Unternehmensseite unterstützt werden.

### Entscheidungen im Team treffen

Die Festlegung der Präsenz-Tage sollte im Regelfall durch die Teams erfolgen. Zur Vermeidung von Konflikten sollten die Teams von Anfang an bei der Entscheidungsfindung durch Schulungs- und Teamentwicklungsformate unterstützt und befähigt werden. Nahezu alle Teams der BSR haben hierzu bereits erste digitale Workshops durchgeführt.

### Befähigung der Führungskräfte in ihrer Führungsrolle

Den erhöhten Aufwand, den viele Führungskräfte durch das Arbeiten im MoA sehen, sollte die BSR durch systematische Unterstützung und Schulung der Führungskräfte berücksichtigen. Dazu braucht es die Vermittlung eines neuen Führungsverständnisses, das auf Vertrauen und Ergebnisorientierung basiert (Pruisken et al. 2020). Des Weiteren sollten die Führungskräfte gezielt auf das Führen auf Distanz geschult werden (Hofmann et al. 2020), und über die Erfahrungen sollte ein kontinuierlichen Austausch zwischen den Führungskräften, z. B. durch Formate wie die kollegiale Beratung, etabliert werden. Die BSR verfügt bereits über ein entsprechendes umfangreiches Fort- und Weiterbildungsangebot und bietet ihren Führungskräften Coaching an.

### Befähigung der Beschäftigten

Mobiles Arbeiten bedeutet, dass die Beschäftigten in der Gestaltung ihrer Arbeit grundsätzlich freier sind und sich daher in stärkerem Maße selbst organisieren und strukturieren können müssen (Mander et al. 2021). Außerdem ist die Kommunikation aufwändiger, und die Anforderungen an die digitalen Kompetenzen sind höher. Um die Beschäftigten der BSR für das langfristige Arbeiten im MoA zu rüsten, sind daher vielfältige Schulungen und unterstützende Maßnahmen erforderlich. Neben der Schulung von digitalen Kompetenzen ist ein Auf- oder Ausbau von Selbstmanagement und Kommunikationsfähigkeiten unabdingbar. Ein weiterer Schwerpunkt bei den begleitenden Maßnahmen sollte die BSR auf die Befähigung zum gesundheitsgerechten Verhalten legen, wie Hofmann et al. (2020) empfehlen.

### Passende IT-Infrastruktur und digitale Befähigung der Beschäftigten

Die BSR muss den Beschäftigten die jeweils benötigte IT-Infrastruktur zur Arbeit im MoA zur Verfügung stellen und entsprechende Schulungen anbieten. Dazu gehört neben der Hardware auch die geeignete Ausstattung mit digitalen Kommunikationstools und Kollaborationsanwendungen (Kauffeld et al. 2016). Letztere sind zwingend erforderlich, um den Aufwand der Zusammenarbeit, der bei mobilem Arbeiten als größer angesehen wird, auszugleichen. Wichtig dabei ist eine effektive Nutzung dieser Anwendungen und Tools für die virtuelle Kommunikation und den Informationsaustausch (Kauffeld et al. 2016).

### Desksharing nutzen, um neue Raumkonzepte zu gestalten

Die Bereitschaft von vielen Beschäftigten, auf einen festen Arbeitsplatz zu verzichten, sollte die BSR für die Etablierung von Desksharing-Modellen nutzen. In einem zukünftigen Raumkonzept sollten flexible und kreative Räume eingeplant werden, die sich für kollaboratives Arbeiten in Teams und zum sozialen Austausch eignen.

### Veränderung der Unternehmenskultur

Als Grundlage für das langfristige Gelingen des mobilen Arbeitens bedarf es einer Änderung der Unternehmenskultur, weg von der Präsenzkultur hin zur Homeofficekultur, die durch starkes Vertrauen in die Beschäftigten, eine wertschätzende Kommunikation sowie Regelungen bezüglich der Erreichbarkeit und der Arbeitsaufgaben im Homeoffice gekennzeichnet ist (Neumann et al. 2020). Die BSR sollte vertrauenserweckende Strukturen und institutionelle Angebote schaffen, die nicht nur formal existieren, sondern auch verlässlich gelebt werden. Dazu gehören Fort- und Weiterbildungsangebote, das Vorhandensein von Karrierewegen, Angebote zur Work-Life-Balance, aber auch Mechanismen zur Selbstkontrolle und -evaluation, wie Feedbacksysteme und betriebliches Vorschlagswesen (Jäckel 2020).

## Schlussbemerkung

Mobiles Arbeiten ist aus der neuen Arbeitsorganisation der BSR nicht mehr wegzudenken. Die Pandemie hat wie ein Transformationsbeschleuniger gewirkt. Was vor zwei Jahren, gerade in einem öffentlichen Unternehmen wie der BSR, noch für keinen vorstellbar war, ist heute Realität. Es wird kein Zurück mehr zu einer Fünf-Tage-Woche im Büro geben. Die Beschäftigten wollen einen relevanten Anteil ihrer Arbeitszeit im MoA arbeiten. Dafür sind sogar viele bereit, auf ihren festen Arbeitsplatz zu verzichten. Die hohe Beteiligung an der Befragung hat den Willen der Beschäftigten zur Mitgestaltung gezeigt. Das Interesse am MoA ist teilweise sogar größer als in Studien aus 2020. Damit überwiegen für die Beschäftigten der BSR auch nach zwei Jahren offensichtlich die Vorteile. Aus den Ergebnissen lassen sich klare Empfehlungen für eine neue Arbeitsorganisation unter Einbezug des Homeoffice ableiten. Eine große Herausforderung wird der Wandel von der Präsenzkultur hin zur „Homeofficekultur“ und damit das Leben des „Neuen Normal“ für die BSR sein. Eine erfolgreiche Implementierung wird maßgeblich die Attraktivität der BSR als Arbeitgeber:in, die entscheidend für den Wettbewerb um Fachkräfte jetzt und in Zukunft sein wird, erhöhen.

## Footnotes

1 Der Begriff „MoA“ wird synonym mit dem Begriff „Homeoffice“ verwendet.
